# Experiences in Treating Cleft Palates

**Published:** 1893-06

**Authors:** J. Adams Bishop

**Affiliations:** New York


### EXPERIENCES IN TREATING CLEFT PALATES.[1]

BY DR. J. ADAMS BISHOP, NEW YORK.

I DO not expect to-night to give expression to anything that is new or even to interest some of you in this matter, but only to give a *résumé* of my experiences in treating cleft palates.

It is unfortunate to be born with a life-long deformity, especially for a highly-sensitive person. Disease and deformity come upon the rich and poor alike, and to whatever position they may be born, they feel this burden which they have to carry through life. The rich seek and pay for something that will conceal their deformities, while the poor show theirs in the amphitheatre and by the wayside, that they may receive, from those who can pity them, a small sum of money.

Deformities are not new, but have existed for centuries, and men of education and science, all along the line of these years, have given to us their experiences, which help us in the dental profession to stand well in what is called advanced surgery.

The chief object of the operation for cleft palate is, of course, the improvement of the voice.

Suppose we begin back in 1500 and come down to our day in the chronological order (as may be found in Coles on the "Deformities of the Mouth") of such surgeons and dentists as have given a thought or appliance for the remedying of the cleft palate.

"1552.—Hollerius, in his 'Observ. ad Calcem de Morbis Internis,' proposes to stop the apertures with wax or sponge.

"1565.—Alexander Petronius, in his 'De Margo Gallico,' proposes, when there is but one opening in the palate, to stop it with wax, cotton, or a gold plate, taking care to give to the instruments the same concave form as the roof of the mouth.

"1579.—Ambrose Paré, in his book on surgery, published in Paris, and in the year 1649 translated into English by Thomas

---

[1] Read before the New York Odontological Society, March 21, 1893.

Johnson, proposes that the cavity should be covered over by a gold or silver plate 'made like unto a dish in figure, and on the upper side.'

"1649.—Isaac Guillemeau, in his 'D'Ouvres,' gave a drawing of an instrument similar in form to Ambrose Paré's instrument, but suggested that, as it was not always possible to adapt the plate perfectly to the roof of the mouth, a lining of sponge or lint should be applied, in order to render the closure more complete.

"1653.—Amatus Lusitanus, in his 'Curat. Medic. Centur.,' mentions a boy with diseased cranium and perforated palate, whose voice was restored by means of the gold plate and sponge.

"1685.—Nic. Tulpius, in his 'Observat. Medici,' mentions the same mode of treatment.

"1715.—Garangeot, in his 'Treatise on Instruments,' is the first that we find making any step in advance of his predecessors with regard to the construction of obturators. Describing one, he says, 'This instrument has a stem in the form of a screw, upon which runs a nut. To make use of it, take a piece of sponge, cut in the shape of a hemisphere, with a flat surface; pass the stem of the obturator through the sponge, and fix it by means of the nut. Dip the sponge in water, squeeze it dry, and introduce it through the aperture.'

"1723.—Fabricii Hieronimi, in his 'Chirurgicis Operâtionibus,' recommends sponge, lint, or silver plate, not suggesting any new form of instrument. He is the first, so far as I have been able to examine these old works, who makes specific mention of congenital cleft palate in contradistinction to accidental cleft or perforation.

"1734.—R. Wiseman, sergeant-surgeon to King Charles II., in his chirurgical treatises, gives evidence of having bestowed much thought upon the treatment of the defects of the palate, though he cannot be said to have made much real and practical progress. His novelty in treatment consisted in filling up the cleft with a paste composed of myrrh, sandarac, and a number of other ingredients. His idea was certainly in advance of his time, for by this means a most important end was gained,—that of perfect exclusion of air by its complete adaptation to the margins of the cleft. We are, unfortunately, not informed how this 'paste palate' was kept in position.

"1739.—Heister, in his 'Institutions of Surgery,' suggests the use of 'a gold or silver plate adapted to the perforation, and furnished with a handle or small tube, which, being armed at the top with a sponge, he may thereby exactly close the perforation.'

"1754.—Astruc, in his 'Treatise on Syphilis,' makes the first mention that we have of a silver button to the metallic obturator, in place of the sponge, in order to avoid the unpleasantness arising from the absorption of mucus. This is the first time that the unpleasant feature of the mucus has been mentioned. I will refer again to this later.

"1786.—M. Pierre Fouchard, in his 'Chirurgeon Dentiste,' gives an account of some instruments which show a very great improvement on the forms previously in use; the sponge, as a means of support to the obturator, being substituted by an arrangement of metallic wings, worked into proper position after introduction into the cleft, by means of a hollow stem and nut, which, when screwed down, kept the wings (covered with soft sponge) across the aperture.

"There are descriptions given of others on the same principle, and of one on a then new plan, depending for its support upon ligatures round the canine teeth.

"1820.—The next advance was made by M. de la Barre, who is the first to mention the use of 'elastic gum' in the restoration of the velum and uvula. The artificial palates designed by this gentleman were ingenious in the extreme, but of such a complicated nature that none but a man of considerable mechanical genius could ever hope to be successful in their application. Still, we must bear in mind the great step taken towards the present instruments in use by the introduction of 'elastic gum.'

"1828.—I now come to a consideration of the artificial palates constructed by Mr. Snell, who arrived at much more satisfactory results in his method of treatment than his predecessors could have done, from the fact that he first obtained an accurate model of the mouth, on which he mounted and fitted his obturator,—a point that up to this time is not mentioned, even if it were practised.

"'My method,' he writes, 'of constructing an obturator is with a gold plate accurately fitted to the roof of the mouth, extending back to the os palati, or extremity of the hard palate, a part of the plate about an inch in length being carried through the fissure.'

"A piece of prepared elastic gum is next attached to the posterior part of the plate, where the natural soft palate commences, extending downward on each side as low as the remaining part of the uvula, and grooved at its lateral edges to receive the fissured portions of the velum. A movable velum is placed in the posterior centre of the elastic gum.

"'It is requisite here to mention that the elastic gum should be placed in a gold frame, and not merely fastened to the posterior part of the plate, as it would shrink up by remaining in the mouth. This frame should pass round its edges only, leaving the centre open. The anterior lateral edges should be made to come considerably over the sides of the fissure, which will prevent their slipping behind it during their altered positions, the whole apparatus being held up by elastic gold springs round the teeth on each side.' "

After seventeen years' study of the above and all that had gone before, Mr. Stearns, a surgeon, of London, in 1845 communicated four articles to the *Lancet* on congenital deficiency of the palate, when he gave a description of an instrument which he had contrived for the treatment of these cases. It was in some respects like the obturators of De la Barre and Snell, though more difficult to construct than either of them. It consisted of a gold plate fitted across the hard palate, having attached to it, by means of two spiral springs, an artificial velum of elastic rubber, consisting of a body, wings, and grooved edges to receive the margins of the cleft. I shall refer again later to Mr. Stearns.

"1857.—Mr. Sercombe, who had for some time paid a great deal of attention to the treatment of the cleft palate, in this year gave a description of the instrument he uses in remedying this defect, in a paper which he read before the Odontological Society, entitled 'Cleft Palate: Its Surgical and Mechanical Treatment.'

"In 1862, Mr. Williams exhibited the following case, showing his mode of treatment: Case of complete fissure of the hard and soft palate, the fissure extending through the whole of the hard palate and uvula.

"1864.—In this year Dr. Norman Kingsley, of New York, brought before the Odontological Society of Great Britain a method of treatment that for its merit demands the highest praise. The instrument itself was not altogether new in form, being to some extent very similar to that which had been constructed some years before by Mr. Stearns.

"The interest attaching to the paper was rather the account of the *modus operandi*, which was briefly mentioned, the two great novelties in Dr. Kingsley's treatment consisting in taking an impression of the parts in plaster of Paris instead of wax, and preparing the elastic rubber vela in metallic moulds, rendering duplication of them a very easy matter.

"1867.—Mr. George Parkinson, in a communication to the *Lancet*, describes his method of treating cleft of the hard and soft palate, which I will not detain you to repeat.

"Next we have an obturator made by Dr. Suerson, of Germany, entirely of hard rubber. A gold medal was presented to this gentleman, on account of his invention, by the Central Association of German Dentists.

"I have endeavored, briefly, it is true, to trace, from the first accounts given, the successive stages by which we have arrived at the present mode of treatment, showing the development of the principle that the obturator should not simply fill up the gap in a cleft palate, but be so constructed as to work on physiological principles with the natural movements of the sides of the cleft.

"In 1844 Sir William Fergusson demonstrated the precise action of the muscles of the split palate; and in 1845 Mr. Stearns gave to the profession an account of an instrument which, from the movements it was capable of, I am led to conclude was constructed with a view to utilize the peculiar muscular action which the year before had been shown to exist by the first-mentioned gentleman.

"This may have been simply accidental, but it is worthy of note."

Dr. Stearns's life commenced about eighty years since, badly handicapped by a deformity of the palatine fissure (with very little, if any, hope of being helped by the surgeon or dentist), and few of our dentists know with what skill and perseverance he struggled to overcome the difficulty which beset his life. His great aim was to perfect his speech and remove this great impediment. Without power to perfect his thoughts in a clear, pure tone, either in his family circle or in the refined and cultivated society which surrounded him, we can well imagine the mortification which led him to choose a life of scholarly habits, and devote much time to study and invention, in the hope of finding some remedy for his misfortune.

At twenty a graduate of Yale, at twenty-three he had a medical diploma, and five years later was in London, presenting to eminent medical authorities there his methods of correcting cleft palate with mechanical appliances. Eminent men in Paris and London recognized his achievements, and advised him to make a specialty in medical practice. This was in August, 1845 (*vide* London *Lancet* of that month). Dr. Stearns was able to put himself on record in this department at this early date in the use of vulcanizable rubber as a material, and to him belongs the priority of this discovery so clearly that the dental profession made no claims to it in the compilation of its literature on this subject up to the centennial year of 1876. Dr. Stearns, however, early discovered that plastic surgery was but partially successful in treating cases of palatine fissures by staphylorrhaphy, nor could ordinary surgeons do it at all by the new method, because incapable and unfitted by want of mechanical skill and experience to undertake the construction and introduction of such appliances. So he very wisely turned his attention to the dental surgeon as being better qualified to furnish the elaborate and difficult mechanism requisite to the wants of the cleft-palate patient. Hence, on his return to America, we find him offering the opportunity to dentists who might wish to perfect themselves in this important department of science, to consult him, at No. 95 Cliff Street, New York, for that purpose. A number of our dentists did avail themselves of this privilege, becoming thus more familiar with his plans and ideas, and have ever since been able to hold leading positions in this department.

His generosity in giving freely his hard-earned experience to the dental profession in the interest of unfortunate cleft-palate patients deserves our thanks and gratitude.

I would introduce here the first three cases in which I made use of an obturator made entirely of hard rubber.

1. In November, 1869, I placed in the mouth of a lady one of these obturators, which has been constantly worn to date. Models of the mouth and obturator were at the International Exhibition at Philadelphia in 1876, and a history of the case was printed in the *New York Medical Journal* in 1878. The obturator was made of hard rubber, and the former defect in the speech would not now be noticed under most circumstances. One peculiar feature of the case is that the lady always retains the obturator in the mouth at night, as well as by day, and claims that its presence greatly softens the air as it passes down into her lungs, though I cannot explain how that can be.

2. In October, 1871, a gentleman about fifty years old came to me, after he had availed himself of all the instruments introduced into the profession up to that time, and was therefore fully competent to judge of the worth of this one. He pronounced it the most comfortable he had used, and said that it enabled him to articulate with ease and distinctness.

3. Dr. Gurdon Buck, in his "Reparative Surgery," published in 1876, reports a case (folio 148) treated by me in October, 1874. In concluding, he says, "The plate, when finally adapted, was worn with much comfort, and the characteristic defect of articulation, which had existed in a marked degree, was almost entirely corrected."

I would now like to call your attention to four distinctive features of this plate: first, the simplicity of its construction, which allows of its being made and adjusted in a short time, compared with a velum instrument; second, the plate being made of one piece, and having a good support in the mouth, sustains all the soft parts in the action of talking, eating, and drinking; third, the heat of the mouth has no effect on it as on the soft velum, which after being worn a month loses its form; fourth, another point in its favor is the ease with which it is kept clean, the finely-polished surface causing the mucus, etc., to pass off from it without remaining as it does on the soft velum.

Dr. Gunning saw these obturators, and was free to criticise them. His description of them can be found in his "Hard Rubber Appliances for Congenital Cleft Palate," published in New York in 1879.

There being no forward action whatever of the superior constrictor muscles, a rigid plate can be worn without intermission, not only in comfort, but with improved condition of the mucous membrane, which is covered in, and of the general health, the nose being as free for breathing as in a normal condition of the parts, while the plate also enables the wearer to utilize the muscles of the cleft velum. The palate is easily made, and, being of hard rubber, does not deteriorate in the mouth. It is not supported by any part of the cleft, and may thus be worn from early childhood without injury to the parts; in fact, its support may even lessen the cleft.

The plate, which is held up by the teeth against the hard roof of the mouth, extends up into the cleft and thence to the back of the pharynx near the tubercle of the atlas, the end being rounded to allow the sides of the pharynx to close during the act of swallowing. This extension into the cleft being spread out over the soft parts on each side, the ununited muscles draw up against it and close off the nasal cavity. The vowel sounds are therefore preserved from the resonance of the nose by the natural action of the muscles, while the nasal sounds are used when necessary, and the tongue is able to form all the lingual consonants, the stiffness of the hard rubber affording the best possible substitute for the muscular firmness of the natural soft palate. To apply this palate, a simple impression of the hard palate and teeth, as is usually taken for the setting of artificial teeth, is quite sufficient, the extension into the soft palate being made by fitting the gutta-percha pattern to the parts without subjecting the patient to the annoyance of obtaining a plaster impression of these sensitive and mobile organs.

This palate is consequently so simple that any accomplished dentist can apply it, and the patient is therefore comparatively independent.

Early use of this artificial palate prevents unnatural action of the tongue, such as attempts to close the cleft with the tongue when the latter should be free to act in articulation, whether in speaking or singing.

Although, as I think has been shown by the foregoing, these obturators are very simple and are of the least possible inconvenience, still I should not advise placing them in the mouths of children under fifteen years of age. In the first place, a child does not appreciate the great advantage secured by the improved speech, and is thus averse to even the slight inconvenience.

In the second place, the fact that the parts affected are constantly growing renders the insertion of the obturator a matter attended by considerable risk of interfering with the normal development.

In case of simple cleft of the soft palate, I am inclined from my observations to believe that staphylorrhaphy will accomplish nearly as good results as an obturator, and will, of course, do away with the inconvenience of a plate.

I know that there are surgeons in this city constantly performing this operation, and, I am told by one of them, successfully.

I had hoped to-night to give you the details of such an operation which was performed long ago, but the sensitiveness of the patient has prevented my obtaining all the information, so I shall have to leave it until some future opportunity.

